# Analysis of diagnosing neonatal respiratory distress syndrome with lung ultrasound score

**DOI:** 10.12669/pjms.38.5.5202

**Published:** 2022

**Authors:** Lie Huang, Dan Ye, Jianhui Wang

**Affiliations:** 1 Lie Huang, The First People’s Hospital of Yinchuan, Yinchuan, China; 2 Dan Ye, The First People’s Hospital of Yinchuan, Yinchuan, China; 3 Jianhui Wang, Children’s Hospital of Chongqing Medical University, Chongqing, China

**Keywords:** Pulmonary ultrasound score, Newborn, Respiratory distress syndrome

## Abstract

**Objectives:**

To investigate the diagnostic effect of lung ultrasound on neonatal respiratory distress syndrome (NRDS) and to analyze the clinical application value of pulmonary ultrasound score.

**Methods:**

Sixty-five NRDS babies who were diagnosed in our hospital from August 2019 to October 2020 were selected as the observation group, and 65 healthy babies were selected as the control group. Children in the two groups underwent lung ultrasound examination. The characteristic signs of lung ultrasound in the two groups were analyzed, and the detection rate of various signs and lung ultrasound score were compared between the two groups.

**Results:**

The main manifestations of lung ultrasound in NRDS children were pulmonary parenchyma, abnormal pleural line, blurred or disappeared A line, pleural effusion, white lung, and weak or disappeared pulmonary pulsation; the lung ultrasound scores of different positions in the observation group were higher than those in the control group, and the differences were statistically significant (P<0.05). The detection rates of signs such as lung parenchyma, abnormal pleural line, disappeared A line, diffuse pulmonary edema, and air bronchogram in the observation group were significantly higher than that in the control group (P<0.05), and there was no significant difference in the detection rate of the sign of B line existence between the two groups (P>0.05).

**Conclusion:**

Lung ultrasound has a high diagnostic value in diagnosing NRDS, and lung ultrasound score can evaluate the severity of NRDS in babies to further optimize the diagnosis results, which has important clinical significance.

## INTRODUCTION

Neonatal respiratory distress syndrome (NRDS), a common neonatal disease, is mainly caused by the immature lung structure and function induced by the lack of pulmonary surfactant (PS), which can further induce hyperventilation, acidosis, and severe hypoxemia.^1^,^2^ The clinical presentations of NRDS are respiratory failure, dyspnea, and cyanosis. A high incidence of NRDS has been found in premature infants, with the characteristics of acute onset and high severity resulting in need of critical care and high mortality.^3^ The natural course of NRDS is the onset of symptoms either at the time of birth or within 12 hours after birth and if not treated in time, infants may die of hypoxia and respiratory failure.^4^ Some data show that the shorter the gestational age of newborns is, the higher the incidence of NRDS is. The incidence of NRDS in newborns with a gestational age of 28 weeks can reach 70%-93%. In recent years, with the liberalization of the two-child policy and the increase of the elective cesarean section rate, the clinical incidence of NRDS has a significant upward trend.^5^ Therefore, identifying and diagnosing NRDS in early stage and taking effective treatment measures to reduce neonatal mortality has important significance to reduce neonatal death rate.

The current diagnostic tools for NRDS include clinical symptom assessment, blood gas analysis and chest X-ray, but the low specificity of these tests may lead to misdiagnosis. Moreover, as the neonatal organs which are not fully developed and are more sensitive to X-rays, repeated examinations can cause and amplify radiation damage.^6^ Therefore, many researchers have been exploring a safer and effective method for the diagnosis of NRDS.

With the development of medical technology, ultrasound diagnosis technology has become a research hotspot, and lung ultrasound (LUS) with a high accuracy has been widely used in clinical diagnosis.^7^ LUS reflects the lung parenchymal lesions by producing different ultrasound artifacts based on the changes of alveolar and interstitial water content, which has been widely used in the diagnosis of many diseases such as pulmonary edema, acute lung injury, and neonatal lung disease. Studies have shown that LUS examination immediately after birth can find NRDS earlier than clinics, and its characteristics of no radiation and repeatable examination beside the bed haves important values for clinical diagnosis and prognosis evaluation.^8^ Li et al. believed that LUS score could evaluate the changes of lung ventilation area in patients with acute respiratory distress syndrome to judge the severity of the disease,^9^ which suggested high values in predicting the prognosis and mortality of patients. Gregorio-Hernández et al. considered that LUS score could provide evidence for early diagnosis and prognosis prediction of NRDS children to guide clinical treatment.^10^ Based on this, we selected 65 cases of NRDS and 65 cases of non-lung disease who received treatment in our hospital in the same period and underwent LUS to explore the diagnostic value of LUS in NRDS and use of LUS score. The present work aims to provide some reference for the selection of clinical diagnosis method of NRDS.

## METHODS

Sixty-five babies with NRDS who were diagnosed in our hospital from August 2019 to October 2020 were selected as the observation group.

### Inclusion and Exclusion criteria:

The inclusione criteria included having NRDS symptoms and acute onset, showing typical abnormalities on chest X-ray films, showing hypercapnia and hypoxemia and mean arterial oxygen partial pressure (PaO_2_)/fraction of inspired oxygen (FiO_2_) < 300 mmHg in arterial blood gas (ABG) analysis, and having an informed consent form signed by baby’s family members. The exclusion criteria included gestational age larger than 42 weeks or smaller than 28 weeks at birth, having breathing difficulties caused by other reasons, and having been treated by pulmonary surfactant or some necessary measures. Another sixty-five healthy babies were selected as the control group. This study was approved by the ethics committee of our hospital (No. 202105016 approved on 17-05-2021), and informed consent was signed by the parents or guardians of the children.

### Lung ultrasound:

Mindray M9 portable color Doppler ultrasound diagnostic instrument was used.

**Fig.1:**
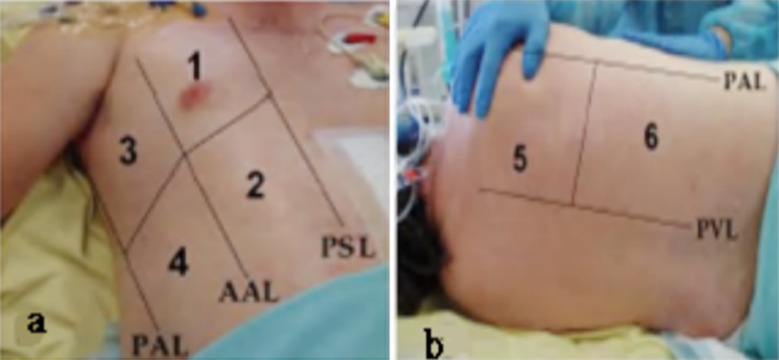
Pictures of area divisions for LUS.

The frequency of the high linear array probe was 10 ~14 MHz. The baby took supine position, lateral and prone position. Chest wall on each side was divided into six areas (Fig.1), anterosuperior, anteroinferior, supraaxillary, sub axillary, posterosuperior, and posterinferior by the parasternal line, anterior axillary line, posterior axillary line, and double nipple connecting line. The probe started from the area below the collarbone to transversely scan along the intercostal space and then longitudinally scan perpendicular to the rib. The examination results were diagnosed by pulmonary ultrasound physician.

### Ultrasound scoring criteria:

Normal LUS shows smooth and clear pleural lines and hyperechoic A-lines equidistant from the pleural lines, with B-lines starting from the pleura and extending deeper when the alveolar and interstitial water content increases.^11^ The 12-zone ultrasound sonogram of the two lungs was scored according to the pulmonary ultrasound scoring method of NRDS:^12^ three points for showing hepatization of lung tissue, i.e., having lung consolidation and disappearance of gas content, two points for the fused B line occupying all intercostal spaces, i.e., having alveolar edema and severe reduction of gas content, one point for showing the fused B line was below the 50% of the scanned intercostal space or multiple isolated B lines, i.e., having focal pulmonary edema, interstitial lung syndrome or subpleural consolidation and moderate reduction of gas content, and zero point for normal livers and normal gas content. The total score was 36 points; the higher the score, the more serious the lung injury.

### Observational index:

The results of the observation group and the control group were compared, and the LUS scores of the two groups at baseline for diagnosis and after treatment were also compared. LUS related indicators used were lung consolidation, pleura line abnormality, air bronchogram, fused B line, alveolar interstitial syndrome, and pleural effusion.

### Statistical Analysis:

SPSS 21.0 was used for statistical analysis of the data. The counting data were expressed by rate and analyzed by x^2^ test. The measurement data were expressed by Mean±SD and analyzed by t-test. P<0.05 was taken statistically significant.

## RESULTS

**Fig.2:**
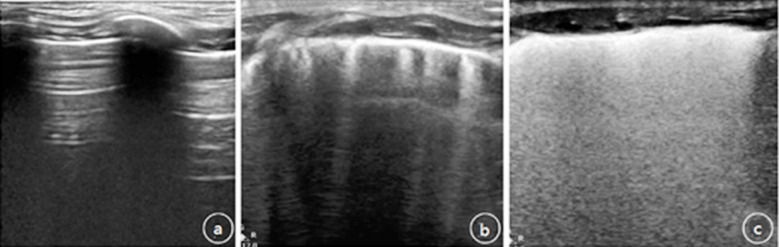
Lung ultrasound results of babies in two groups.

The gender, gestational age, birth weight and mode of delivery in the two groups had no statistical significance (P>0.05) (Table-I). In the control group, the pleural line was clear and smooth, both lungs were A-line, only sporadic B-line was shown in the outer zone, lung sliding existed, no adenocarcinoma in situ (AIS) and pleural effusion were found (Fig.2a).

**Table I:** Comparison of general data between the two groups of children.

Group	Gender (male/female)	Gestational age (week)	Birth weight (kg)	Mode of delivery (vaginal/caesarean section)
Observation group	36/29	31.36±3.02	1.77±0.56	22/43
Control group	33/32	30.81±2.86	1.72±0.57	26/39
X^2^/t	0.186	0.882	0.563	0.421
P	>0.05	>0.05	>0.05	>0.05

The observation group showed diffuse lesions, disappearance of A line, thickened, blurred, or even disappeared pleural line, visible AIS, or even white lung (Fig.2b and 2c). The LUS score of different parts (both lungs, left lung, right lung, bilateral lung, and base of lungs) in the observation group was significantly higher than that in the control group, and the difference was statistically significant (P<0.05, Table-II).

**Table II:** Comparison of lung ultrasound scores between the two groups.

Group	Both lungs	Left lung	Right lung	Bilateral lung	Base of lungs
Observation group (mean±SD, point)	24.77±6.96	9.36±2.86	9.12±1.12	10.89±2.25	5.17±1.02
Control group (mean±SD, point)	11.90±5.85	7.03±1.85	5.14±1.11	5.94±1.26	3.21±0.69
t	9.479	4.634	16.653	12.824	10.855
P	<0.05	<0.05	<0.05	<0.05	<0.05

The detection rate of signs such as lung parenchyma, pleural line abnormality, disappearance of A line, diffuse pulmonary edema, and air bronchogram in the observation group was significantly higher than that in the control group (P<0.05). There was no significant difference in the detection rate of existence of B line between the two groups (P>0.05, Table-III). The sensitivity and specificity of lung parenchyma, pleural line abnormality, and disappearance of A line were 100.0%. The sensitivity and specificity of lung parenchyma, pleural line abnormality, and air-bronchogram sign were 80.0% and 100.0% respectively.

**Table III:** Comparison of detection rates of various signs between two groups [n (%)].

Lung ultrasound parameters in both groups						

Group	Pulmonary parenchyma	Disappearance of A line	Diffuse pulmonary edema	Abnormal pleural line	Air bronchogram	Existence of B line
Observation group	65 (100.0)	65 (100.0)	48 (73.8)	65 (100.0)	52 (80.0)	6 (9.2)
Control group	0 (0.0)	0 (0.0)	0 (0.0)	0 (0.0)	6 (9.2)	12 (18.5)
X^2^	85.045	85.045	53.106	85.045	43.268	1.528
P	<0.05	<0.05	<0.05	<0.05	<0.05	>0.05

## DISCUSSION

The results of this study showed that all children with NRDS had a sign of lung parenchyma, which is similar to the research of Liu et al.^13^ This study also found that the detection rates of lung parenchyma, abnormal pleural line, disappearance of A line, diffuse pulmonary edema, and air bronchogram in children with NRDS were significantly higher than those in children without lung disease, but there was no statistical difference in the detection rate of existence of B line between the two groups, suggesting that the detection performance of LUS was good. A normal lung pleural line is a smooth, clear, regular curve with a width of no more than 0.5 mm; it is abnormal if it becomes rough, membranous or irregular, with a thickness > 0.5 mm. Diffuse pulmonary edema is another characteristic sign. Conventional X-rays only show uniform, bilateral, glassy changes, making it difficult to determine whether the cause of a lesion is pleural effusion, pulmonary edema or atelectasis; however, LUS provides additional clinical information and for differential diagnosis. Other characteristic signs such as lung parenchyma, disappearance of the A line, presence of the B line and air-bronchogram sign may reflect early lung lesions.^14^-^16^ The alveolar-air interface has surface tension. The lack of surface active substances in NRDS children leads to alveolar compression and poor alveolar ventilation. X-rays show reduced translucency and fine granular shadows in both lungs, and blood flow does not exchange through the compressed area, resulting in lower partial pressure of oxygen and higher partial pressure of carbon dioxide; the compensatory mechanism leads to bronchiectasis.^17^

However, ultrasound has been used as a qualitative analysis before and there was a lack of quantitative indicators to judge the severity of NRDS. Therefore, this study made quantitative scoring on the ultrasound results. The results showed that the ultrasound scores of both lungs, left lung, right lung, bilateral lung, and base of lungs in the observation group were higher than those in the control group, indicating that the LUS score could show the difference between the lungs of normal newborns and NRDS children. Liu et al. also found that there was a sign of lung parenchyma in NRDS children by lung ultrasound,^18^ and the LUS score of NRDS children was significantly different from that of non-lung disease children.

Ultrasound examination of 12 areas of the lung can capture the changes of lesions from the front chest to the back. Therefore, bedside lung ultrasound, as a simple, non-invasive, radiation-free, and flexible examination method, not only broadens the field of vision for clinicians in the selection of NRDS examination methods but also makes up for the low sensitivity of bedside chest film.^19^ In addition, in this study, the sensitivity and specificity of LUS in the diagnosis of NRDS children were high. In the study of Perri et al.^20^ the sensitivity and specificity of LUS in the diagnosis of NRDS children were 86.00% and 88.00%, respectively, which also confirmed that LUS was a noninvasive and repeatable method.

### Limitations of the study:

The sample size of this study was small, which may cause some bias to the results. In the future, the author will carry out randomized multi-center studies with large sample size.

## CONCLUSION

Lung Ultrasound Score (LUS) has obvious diagnostic values for NRDS. LUS has high detection rates for related signs. It has important clinical values for the diagnosis and dynamic real-time observation of NRDS.

### Authors’ Contribution:

**LH & JHW:** Study design, data collection and analysis.

**DY & JHW:** Manuscript preparation, drafting and revising.

**LH & JHW:** Review and final approval of manuscript.

**JW:** Responsible for the integrity and accuracy of the study.
